# Mechanisms of AGE-induced VSMC phenotypic switching and macrophage modulation in human abdominal aortic aneurysms

**DOI:** 10.3389/ebm.2025.10527

**Published:** 2025-08-07

**Authors:** Xiaoying Ma, Jinfang Xu, Huiying Sun, Jiajun Liu, Shibo Xia, Hao Zhang, Chaoyi Cui, Chao Song

**Affiliations:** ^1^Department of Vascular Surgery, Changhai Hospital, Shanghai, China; ^2^Hepatobiliary Surgery Center, Tongji Hospital, School of Medicine, Tongji University, Shanghai, China; ^3^ Department of Health Statistics, Naval Medical University, Shanghai, China; ^4^ School of Pharmacy, East China University of Science and Technology, Shanghai, China; ^5^Department of Vascular Surgery, Shanghai Ninth People’s Hospital, School of Medicine, Shanghai Jiao Tong University, Shanghai, China

**Keywords:** abdominal aortic aneurysms, AGE-RAGE, VSMC phenotypic switch, NLRP3, macrophages

## Abstract

Advanced glycation end products (AGEs) have been associated with vascular pathologies including abdominal aortic aneurysms (AAAs), although their causal role remains unclear. In this study, we observed significant accumulation of AGEs in human AAAs, particularly in cases associated with intraluminal thrombus (ILT). *In vitro*, AGE exposure induced vascular smooth muscle cell (VSMC) migration and suppressed contractility, accompanied by reduced expression of contractile markers (α-SMA and MYH11) and elevated MMP-2. This phenotypic transformation was linked to the activation of the NLRP3 inflammasome and RAGE/RhoA/ROCK signaling, and was reversible upon inhibition of RAGE, RhoA, or ROCK. In macrophages, AGE pretreatment had minimal effects on basal cytokine secretion but attenuated LPS-induced IL-6 and IL-1β release and NF-κB activation. Co-culture experiments further revealed that AGE-pretreated macrophages reduced LPS-driven pro-migratory effects on VSMCs. Spatial transcriptomics demonstrated enriched AGE-RAGE signaling in αSMA+ VSMCs and CD68+αSMA+ macrophage-like VSMCs in ILT-containing AAAs. Overall, these associative findings implicate AGE-RAGE signaling in AAA pathogenesis and warrant further investigation to establish causality.

## Impact statement

This study reports on the association between advanced glycation end products (AGEs) and vascular smooth muscle cell (VSMC) dysfunction/macrophage responses in abdominal aortic aneurysms (AAAs), particularly within the intraluminal thrombus. Using spatial transcriptomics (GeoMx^®^ DSP), we detected the co-localization of AGE-RAGE signaling in VSMC niches and demonstrated *in vitro* that AGE exposure correlates with VSMC phenotypic transformation via the RAGE/RhoA/ROCK pathways, along with the modification of macrophage responses. Pharmacological reversal of AGE-induced changes provides mechanistic hypotheses for AAA pathogenesis and merits future causal studies of the AGE-RAGE axis in vascular remodeling.

## Introduction

Advanced glycation end products (AGEs) encompass a diverse array of irreversible adducts formed through the nonenzymatic glycosylation of proteins, lipids, and nucleic acids by reducing sugars [1]. Normally, AGE formation occurs at a low rate, but accelerates significantly in both microvascular and macrovascular diseases [2]. Vascular smooth muscle cells (VSMCs) are located in the middle layer of arterial vessels, playing a crucial role in maintaining arterial structure and regulating vascular tone. Under normal physiological conditions, VSMCs exhibit a contractile phenotype. However, in response to harmful stimuli, VSMCs lose their contractile properties and transition to a synthetic phenotype. This transformation is pivotal in the development and progression of abdominal aortic aneurysms (AAAs) [3].

Studies have confirmed that AGEs can stimulate the proliferation of VSMCs [4]. The receptor for AGEs (RAGE) is particularly important as it mediates the proliferation and migration of VSMCs induced by AGEs [5, 6]. In response to stress, AGEs accumulate and bind to RAGE with high affinity, triggering oxidative stress, inflammation, and procoagulant responses. These processes ultimately contribute to vascular wall lesions, including remodeling of the aortic vasculature and dysfunction of the aortic endothelium.

The intraluminal thrombus (ILT) within abdominal aortic aneurysms is a complex fibrin structure comprised mainly of canaliculi, platelets, erythrocytes, and other hematopoietic cells [7]. Clinically, ILT is found in 75% of AAA sacs and is associated with arterial wall hypoxia, cellular inflammation, and apoptosis of the extracellular matrix, and can contribute significantly to aneurysm growth, potentially leading to rupture [8–12]. While the AGE-RAGE axis has been implicated in vascular pathologies, its role in ILT-associated AAA remains underexplored. Characterizing this association could offer insights into the mechanisms of aneurysm progression.

The Rho GTPase protein family, which includes RhoA, Rac1, and Cdc42, plays a critical role in cellular functions such as contraction, migration, and the organization of the actin cytoskeleton. Rho-associated kinase (ROCK), a key downstream effector protein of Rho GTPases, phosphorylates various targets to influence these cellular processes. Studies have demonstrated that the RhoA/ROCK pathway is involved in regulating the phenotype of VSMCs induced by platelet-derived growth factor BB (PDGF-BB) [13]. Activation of the Rho/ROCK pathway can lead to the phosphorylation of threonine 696 on the Myosin Phosphatase Target subunit-1 (MYPT1), resulting in abnormal contraction of vascular smooth muscle [14]. Inhibition of the RAGE/RhoA/ROCK signaling pathway has been shown to protect the integrity of the aortic wall [15]. *However, whether Rho GTPases and their downstream signals are implicated in the regulation of VSMC phenotype mediated by AGEs has not been conclusively established.*

The NOD-like receptor family pyrin domain 3 (NLRP3) inflammasome is a key cytosolic complex involved in initiating inflammatory responses. It consists of NLRP3, apoptosis-associated speck-like protein (ASC), and procaspase-1, which together regulate the maturation and secretion of pro-inflammatory cytokines such as IL-1β and IL-18. An increasing number of studies have provided conclusive evidence that the NLRP3 inflammasome can be activated by AGEs [16]. AGEs upregulate the mRNA or protein expression of inflammasome-related molecules (such as NLRP3, caspase-1, and IL-1β) in non-myeloid cells (such as podocytes, nucleus pulposus cells, and placental cells) [17]. Understanding how AGEs may influence VSMC inflammatory responses could elucidate pathways relevant to vascular complications.

This research suggests a potential role for AGEs in the pathogenesis of abdominal aortic aneurysms, particularly by modulating the interactions between vascular smooth muscle cells and macrophages. By characterizing context-dependent responses to AGEs in VSMCs and macrophages, this study provides mechanistic insights into AAA pathophysiology and identifies signaling pathways that warrant further investigation.

## Materials and methods

### Cell culture and reagents

THP-1 cells and HA-VSMC were procured from Pricella Life Science & Technology Co., Ltd. HA-VSMC is a cell line exhibiting fibroblast-like morphology that was isolated from the smooth muscle of the aorta of an 11-month-old white female patient. The cell identification results are shown in Supplementary Figure S1. HA-VSMCs were cultured in F-12K medium (Cat. No. 30-2004, ATCC, Manassas, United states) supplemented with 0.05 mg/ml L-ascorbic acid, 0.01 mg/mL insulin, 0.01 mg/mL transferrin, 10 ng/mL sodium selenite, 0.03 mg/mL endothelial cell growth supplement, 10% FBS, 10 mM HEPES, 10 mM TES, and 1% penicillin/streptomycin. Third- to fourth-generation HA-VSMCs were used in the cell-based experiments of this study. THP-1 cells were maintained in RPMI 1640 medium (Cat. No. SH30809.01, HyClone, MA, United states) supplemented with 10% fetal bovine serum (FBS) (Cat. No. FBSSR-01021-50, Cyagen, CA, United states). Prior to experimentation, THP-1 cells were differentiated using 100 ng/mL phorbol 12-myristate 13-acetate (PMA) Cat. No. P1585, Sigma, MA, United states). Z-VAD-FMK (pyroptosis inhibitor, Cat. No. HY-16658B), CY-09 (NLRP3 inhibitor, Cat. No. HY-103666), FPS-ZM1 (RAGE inhibitor, Cat. No. HY-19370), CCG-1423 (RhoA inhibitor, Cat. No. HY-13991), Y-27632 (ROCK inhibitor, Cat. No. HY-10071) and Lipopolysaccharides (Cat. No. HY-D1056) were all purchased from MedChemExpress LLCNJ, United states. Advanced glycation end product-BSA (AGE-BSA) was purchased from Abcam (Cat. No. ab51995, Abcam, Cambridge, United kingdom).

### Real-time quantitative reverse transcription-polymerase chain reaction (qRT-PCR)

As previously reported [18], total RNA was extracted using the FastPure Cell/Tissue Total RNA Isolation Kit (Cat. No. RC112-01, Vazyme, Nanjing, China). Subsequently, cDNA synthesis was performed using AceQ qPCR SYBR Green Master Mix (Vazyme, Cat. No. Q121-02). For quantitative real-time PCR (qPCR), the HiScript III SuperMix for qPCR (Vazyme, Cat. No. R323-01) was employed, and the reactions were analyzed using a CFX96 Fluorescence quantitative PCR instrument (Bio-Rad, CA, United states). The primer sequences utilized are detailed in Table 1. Gene expression data were normalized to GAPDH as an internal reference and quantified using the 2^−ΔΔCT^ method.

**TABLE 1:** Primer sequences in qRT-PCR.

Primers	Sequences
ACTA2	F: AGTTCCGCTCCTCTCTCCAA R: AACGCTGGAGGACTTGCTTT
MMP-2	F: GAGTGCATGAACCAACCAGC R: GTCTGGGGCAGTCCAAAGAA
NLRP3	F: CTTGGAGACATCCTGTCAGGG R: AGTCACAAGACCAGGCATATTCT
TNF	F: GACAAGCCTGTAGCCCATGT R: GGAGGTTGACCTTGGTCTGG
IL-6	F: TTCGGTCCAGTTGCCTTCTC R: TGTTTTCTGCCAGTGCCTCT
IL1B	F: AACCTCTTCGAGGCACAAGG R: AGATTCGTAGCTGGATGCCG
NOS2	F: GCCATAGAGATGGCCTGTCC R: GGGGACTCATTCTGCTGCTT
GAPDH	F: AATGGGCAGCCGTTAGGAAA R: GCGCCCAATACGACCAAATC

Abbreviations: F, forward; R, reverse.

### Digital space profiling (DSP)

Six biopsies were obtained from AAA patients (3 with ILT and 3 without ILT) from Shanghai Changhai Hospital following approval by the Ethics Committee (approval number: CHEC2022-052). Detailed clinicopathological characteristics can be found in Table 2. Whole-exome sequencing was conducted on specific regions of interest (ROIs) using the GeoMX Digital Spatial Profiling platform, with subsequent data analysis and processing performed using the R language. Differentially expressed mRNA was identified using criteria of adjusted p-value <0.05 and |Log2Fold Change| > 1.

**TABLE 2:** Demographic data of AAA patients with and without intraluminal thrombus.

Patient ID	G1-1	G2-1	G1-2	G2-2	G1-3	G2-3
ILT	Y	N	Y	N	Y	N
Age, years	66	68	73	66	65	71
Sex	Female	Male	Male	Female	Male	Male
AAA diameter, mm	52	54	68	48	65	58
Hypertension	Y	Y	Y	N	Y	N
Diabetes mellitus	N	N	N	N	N	N
Smoking history	N	N	Y	N	Y	Y
CAD	Y	N	N	N	N	N
COPD	N	Y	N	N	N	N
PAD	N	N	N	N	N	N

Abbreviations: AAA, abdominal aortic aneurysm; CAD, coronary artery disease; COPD, chronic obstructive pulmonary disease; PAD, peripheral arterial disease.

### ELISA assay of AGE/CCL2/IL-6/MMP-2

A total of 16 specimens from AAA patients were collected and stored in liquid nitrogen for future experiments. Non-AAA samples (N = 5) were obtained during aorta-bifemoral bypass surgery for aorta-iliac occlusive disease. The clinicopathological characteristics of the AAA patients are detailed in Table 3. Ethical approval for this study was obtained from the Ethics Committee of the Shanghai Changhai Hospital. Aortic tissue lysates were prepared using cell lysis buffer to determine protein concentrations. The competitive AGE-ELISA procedure was conducted using an AGE Assay kit (Abcam, Cat. No. ab238539) to assess advanced glycation end-products. Inflammatory factors, including MMP2, IL-6, and MCP-1, were quantified using specific ELISA kits: MMP2 Human ELISA Kit (Cat. No. KHC3082, Invitrogen, CA, United states), IL-6 Human ELISA Kit (Invitrogen, Cat. No. EH2IL6), and MCP-1 Human Instant ELISA™ Kit (Invitrogen, Cat. No. BMS281INST), respectively. All of these assays were performed according to the manufacturers’ instructions.

**TABLE 3:** Demographic data of AAA patients with and without intraluminal thrombus.

Variables	AAA with ILT	AAA without ILT
No.	8	8
Age, years	68.9 ± 5.6	70.6 ± 4.4
Male patients	8/8	7/8
AAA diameter, mm	66.4 ± 1.0	63.2 ± 0.4
Hypertension	5/8	3/8
Diabetes mellitus	1/8	0/8
Smoking history	5/8	3/8
CAD	1/8	2/8
COPD	0/8	0/8
PAD	0/8	0/8
Antiplatelet therapy	1/8	4/8
Statins	0/8	1/8

Continuous variables are reported as mean ± standard deviation.

Abbreviations: AAA, abdominal aortic aneurysm; CAD, coronary artery disease; COPD, chronic obstructive pulmonary disease; PAD, peripheral arterial disease.

### Western blot

As previously reported [19], the total proteins were separated on a 10% SDS-PAGE gel (Cat. No. PG212, Epizyme, MA, United states) and subsequently transferred onto PVDF membranes (Bio-Rad Laboratories Inc., CA, United States). After blocking with 1x protein-free fast blocking buffer (Epizyme, Cat. No. PS108P) for 1 h, the membranes were incubated overnight at 4°C with various primary antibodies, including a smooth muscle actin Polyclonal antibody (Cat. No. 14395-1-AP, Proteintech, Wuhan, China), NLRP3 Polyclonal antibody (Proteintech, Cat. No. 27458-1-AP), MMP-2 Antibody (Cat. No. 4022S, CST, MA, United states), Phospho-IκBα (Ser32) (14D4) Rabbit mAb (CST, Cat. No. 2859T), and GAPDH (14C10) Rabbit mAb (CST, Cat. No. 2118T). Subsequently, the membranes were incubated with Peroxidase AffiniPure Goat Anti-Rabbit IgG (H + L) (1:10000, Cat. No. FZ111-035-003, Jackson ImmunoResearch Inc., PA, United states) for 1.5 h. Detection of protein bands was performed using the Omni-ECL™ basic chemiluminescence detection kit (Epizyme, Cat. No. SQ202), and imaging was conducted with the ChemiDocTM MP chemiluminescence image analysis system (BIO-RAD).

### Transwell migration assay

VSMCs were treated with AGE-BSA and/or inhibitors. Then 2 × 10^5^ cells in serum-free medium were seeded onto 6.5-mm Transwell^®^ inserts with an 8.0-µm Pore Polycarbonate Membrane (Cat. No. 3422, Corning, NY, United states). The lower chamber contained a culture medium supplemented with 10% FBS to serve as a chemoattractant. After incubation for 24 or 72 h, the cells that had migrated to the underside of the membrane were fixed using 4% paraformaldehyde, stained with 0.1% crystal violet (Cat. No. MS4006-100ML, Maokangbio, Shanghai, China), and subsequently photographed under a microscope. This Transwell^®^ migration assay allows for the assessment of cell migration in response to different treatments, providing insights into the migratory behavior of VSMCs under experimental conditions.

### Immunofluorescence

VSMCs were fixed with 4% paraformaldehyde at room temperature for 15 min, followed by three washes with PBS. Subsequently, they were incubated with 0.3% Triton X-100 at room temperature for 30 min and washed again three times with PBS. The cells were then treated with a 3% hydrogen peroxide solution at room temperature for 25 min in the dark, followed by another three washes with PBS. After blocking with 3% BSA at room temperature for 30 min, the cells were incubated overnight at 4°C with MYH11 Antibody (1:100, Proteintech, Cat. No. 21404-1-AP). Following three washes with PBS, the cells were incubated with an anti-rabbit HRP-labeled secondary antibody at room temperature for 1 h in the dark, again followed by three washes with PBS. A freshly prepared fluorescent chromogenic solution (Cat. No. SD0155, Simuwubio, Shanghai, China) was applied and allowed to develop color for several minutes, with the reaction being stopped with PBS. The cells were then incubated with a DAPI dye solution at room temperature for 10 min in the dark, washed three times with PBS, and finally mounted with an anti-fluorescence quenching mounting medium for observation under a microscope.

### Lactate dehydrogenase (LDH) release assay

LDH release was quantified using a lactate dehydrogenase cytotoxicity assay kit (Cat. No. C0016, Beyotime Biotech, Shanghai, China) following the manufacturer’s instructions. VSMCs were cultured in a 96-well plate until they reached 80–90% confluence. The LDH detection working solution was then added to each well, and the plate was incubated at room temperature in the dark for 30 min. Absorbance was measured at a wavelength of 490 nm using a BioTek microplate reader (H1MFD). This assay provides a quantitative assessment of LDH release, indicating cellular cytotoxicity or membrane damage.

### NF-κB transcription factor assay

Nuclear proteins were extracted using the Nuclear Extract Kit (Cat. No. 40010, Active Motif, Inc., CA, United states). Subsequently, cRel, p52, and p62 levels were assessed using TransAM NF-κB Transcription Factor Assays (Active Motif, Inc., Cat. No. 43296). The experimental protocol proceeded as follows: First, 30 μL of Complete Binding Buffer was added to each well of the TransAM plate, followed by the addition of 10 μg of nuclear protein (diluted to 20 μL). The plate was then incubated at room temperature for 1 h with gentle shaking at 100 rpm. After incubation, each well was washed three times with 200 μL of 1X Wash Buffer. Subsequently, 100 μL of the primary antibody solution (diluted 1:1000) was added to each well and the mixtures were incubated at room temperature for 1 h. Following another set of three washes with 1X Wash Buffer, 100 μL of HRP-conjugated secondary antibody solution (diluted 1:1000) was added to each well and the mixtures were incubated at room temperature for an additional hour. After a final round of washing, 100 μL of Developing Solution was added to each well, which was incubated at room temperature in the dark for 5 min. The reaction was terminated by adding 100 μL of Stop Solution to each well, and the absorbance was measured at a wavelength of 450 nm using a BioTek microplate reader (H1MFD). This procedure enabled the quantification of NF-κB transcription factors (cRel, p52, and p62) bound to DNA in the nuclear extracts, providing insight into NF-κB activity in the samples.

### ELISA detection of cytokines

After incubating the cells with different concentrations of advanced glycation end products (AGEs) for 24 h, 1 μg/mL of lipopolysaccharide (LPS) was added for an additional 4 h. The cell culture supernatant was then collected and centrifuged at 10,000 rpm for 5 min. Subsequently, the levels of IL-6 and IL-1β were quantified using the QuantiCyto^®^ Human IL-6 ELISA kit, (Cat. No. EHC007.96, NeoBioscience, Shenzhen, China) and the human IL-1β ELISA kit (Abcam, Cat. No. ab214025), following the respective kit instructions. These assays were employed to assess the secretion of IL-6 and IL-1β cytokines under these experimental conditions.

### Cell viability assay

VSMCs were seeded in 96-well plates at a density of 5000 cells per well. After 4 h of incubation, 200 μg/mL of advanced glycation end products (AGEs) was added to each well. Cell viability was assessed using the Cell Titer-Glo 2.0 Cell Viability Assay (Cat. No. G9242, Promega, WI, United states) from days 1–5 following treatment. This assay measures cellular ATP levels as an indicator of cell viability and metabolic activity over the specified time course.

### NFκB dual luciferase reporter assay

The 293FT cells in the logarithmic growth phase were adjusted to a density of 4 × 10^5^ cells/mL and seeded into a 6-well plate. The cells were incubated overnight at 37°C in a 5% CO2 incubator. To transfect the cells, 2 µg of the NFκB Luciferase Reporter Plasmid (Cat. No. 11501ES03, Yeasen, Shanghai, China) was diluted in 125 µL of Opti-MEM medium and mixed gently. Similarly, 10 µL of Lipofectamine 2000 (Invitrogen, Cat. No. 10668018) was diluted in 125 µL of Opti-MEM medium, mixed gently, and incubated at room temperature for 5 min before being combined with the plasmid DNA. The mixture was allowed to stand for an additional 20 min. The resulting complex (250 µL) was added to each well, and the cells were incubated for 24 h. After transfection, the cells were adjusted to a density of 2 × 10^5^ cells/100 µL and seeded into a 96-well plate, and incubated overnight at 37°C in a 5% CO2 incubator. In 293T cells, AGEs were used to activate the NFκB signaling pathway. Through concentration screening, the EC50 was determined to be 52.7 µg/mL (Supplementary Figure S2), and a concentration of 100 µg/mL was selected to activate the NFκB signaling pathway. The test compounds (FPS-ZM1/CCG-1423/Y-27632) were prepared at the following final concentrations: 1, 10, and 10 µM (4-fold serial dilutions, with 9 concentration points in total). After 2 h of treatment, 100 µg/mL of AGEs was added, and the cells were co-incubated for 24 h. Dual luciferase reporter gene assays were performed following the instructions provided in the Dual Luciferase Reporter Gene Assay Kit (Yeasen, Cat. No. 11402ES60) and using an Envision 2105 multimode plate reader (PerkinElmer). The results were calculated using the following formula: 1). Experimental Group Ratio = (Experimental Group Firefly Luminescence (F) - Background Firefly Luminescence (F))/(Experimental Group Renilla Luminescence (R) - Background Renilla Luminescence (R)); 2). Control Group Ratio = (Control Group Firefly Luminescence (F) - Background Firefly Luminescence (F))/(Control Group Renilla Luminescence (R) - Background Renilla Luminescence (R)); 3). Fold Change = Experimental Group Ratio/Control Group Ratio.

Note:

a) Background F: Untransfected cells + Firefly luciferase assay reagent
b) Background R: Transfected cells + Firefly luciferase assay reagent + Renilla luciferase assay reagent
c) Experimental Group: Transfected cells treated with compounds
d) Control Group: Transfected cells without treatment, used for standardization of results

### Caspase-1 activity assay

The enzymatic activity of caspase-1 was assayed using a Caspase-1 Colorimetric Assay Kit (MCE, Cat. No. HY-K2610-100T) according to the manufacturer’s protocol. Absorbance was measured at 405 nm by using a plate reader.

### Statistical analysis

The data were analyzed using GraphPad Prism 8.0 software (GraphPad Software Inc., USA) and are presented as the mean ± standard deviation (SD). Statistical significance was determined using a One-way ANOVA followed by a Dunnett’s multiple comparisons test. A p-value <0.05 was considered statistically significant.

## Results

### AGE effects on human VSMCs: promotion of migration and inhibition of contraction

**FIGURE 1:**
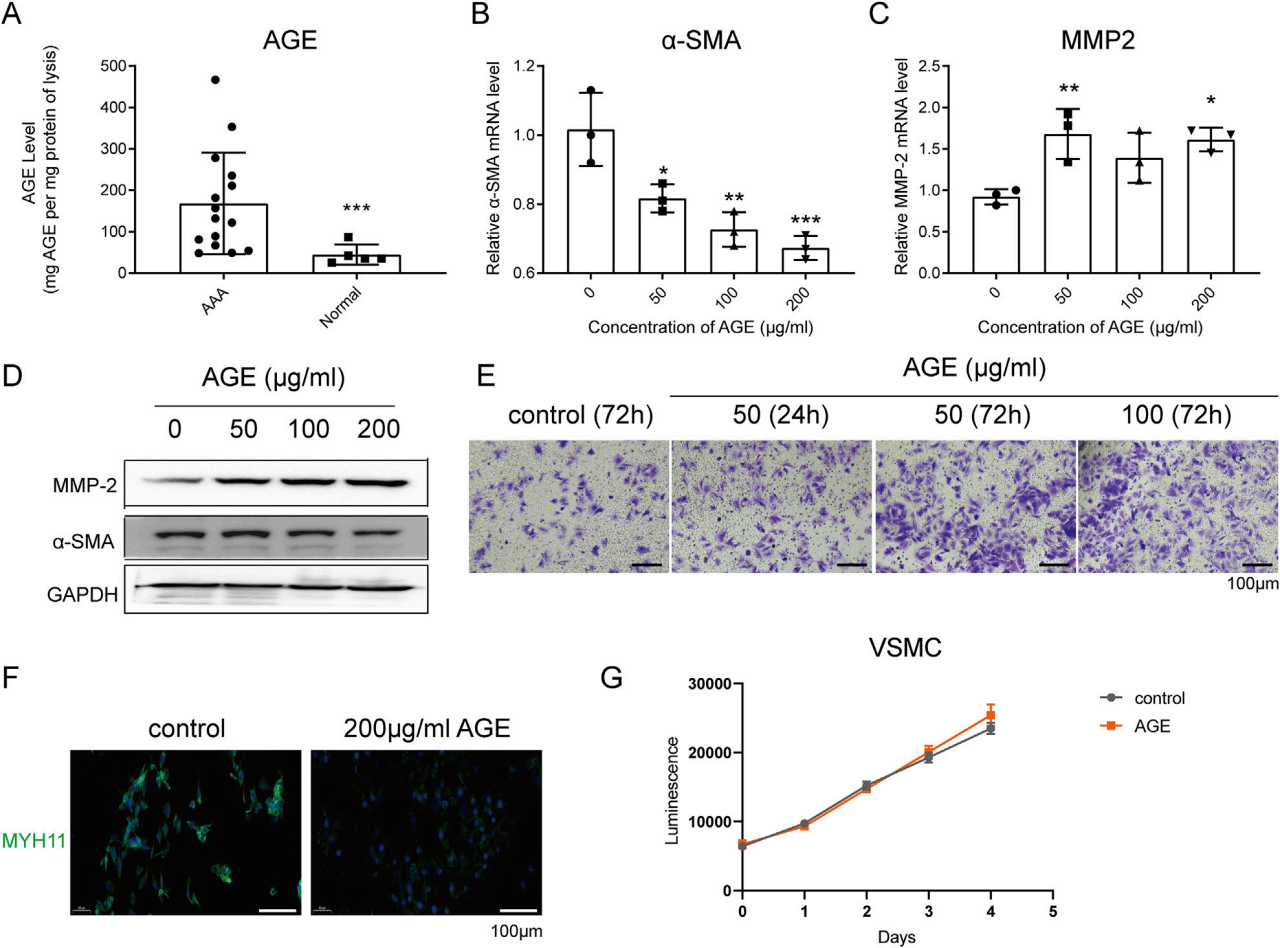
AGE Effects on Human VSMCs: Promotion of Migration and Inhibition of Contraction. **(A)** ELISA analysis detected the accumulation of AGEs in lysates of human aortic tissues. **(B–D)** RT-qPCR **(B,C)** and Western blot **(D)** detected the expression of the contraction marker α-SMA and the migration marker MMP-2 in human VSMCs after AGE treatment for 24 h; **(E)** Transwell migration experiment detected the migration of human VSMCs after AGE treatment for 24 h or 72 h; **(F)** Immunofluorescence detected the expression of the contraction marker MYH11 in human VSMCs after AGE treatment for 24 h. Blue fluorescence indicates DAPI staining, and green indicates MYH11 staining. **(G)** The CTG assay detected the growth curve of human VSMCs under the treatment with 200 μg/mL AGE. Data are presented as the mean ± SD from three independent experiments and were analyzed with a one-way ANOVA followed by a Dunnett’s multiple comparisons test. Asterisks indicate significant differences (*P < 0.05, **P < 0.01, ***P < 0.001) between the treatment group and the control group.

We initiated our research by investigating whether AGE expression differs between AAA specimens and normal arteries. ELISA assays revealed a significant increase in AGE accumulation in human AAA specimens (Figure 1A). RT-qPCR and Western blot analyses revealed that AGEs, administered at varying concentrations (50, 100, 200 μg/mL), significantly suppressed the expression of the contraction marker α-SMA, while concurrently enhancing the expression of the migration marker MMP-2 in human VSMCs following a 24-h treatment period, exhibiting a concentration-dependent response (Figures 1B–D). Results from the Transwell migration assay demonstrated that compared to VSMCs treated with AGEs for 24 h, a higher number of cells migrated following a 72-h treatment (Figure 1E). Immunofluorescence analysis further confirmed that AGEs (200 μg/mL) markedly attenuated the expression of the contraction marker MYH11 (Figure 1F). However, CTG results demonstrated that higher concentrations of AGEs did not significantly promote VSMC proliferation, contrary to previous reports [20, 21] (Figure 1G).

### AGE-mediated transformation of VSMCs from contractile to migratory phenotype via NLRP3 activation

**FIGURE 2:**
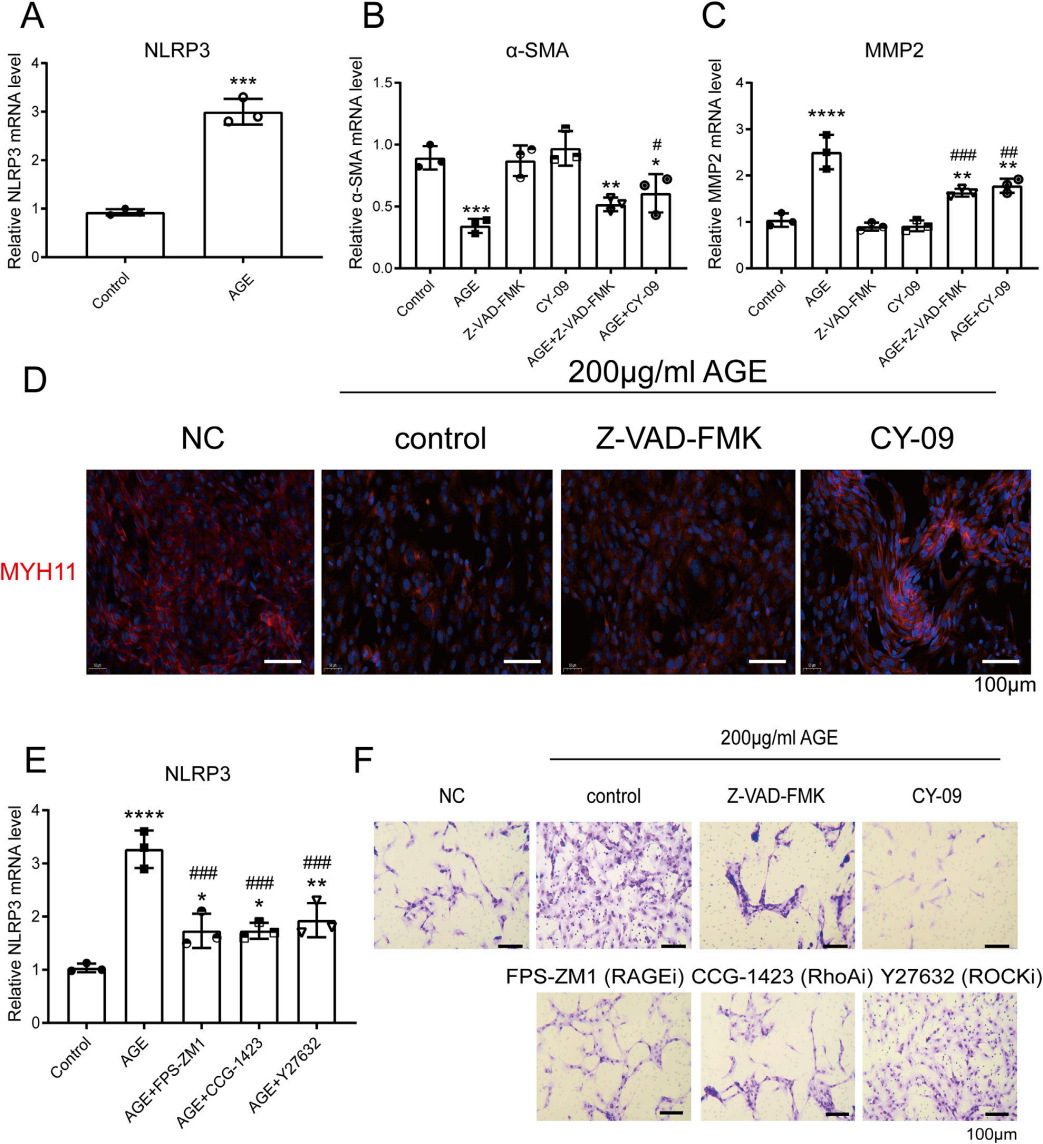
AGE-Mediated Transformation of VSMCs from Contractile to Migratory Phenotype via NLRP3 Activation. **(A)** RT-qPCR detected the expression of NLRP3 in human VSMCs after AGE treatment for 48 h; **(B,C)** RT-qPCR detected the expression of α-SMA **(B)** and MMP-2 **(C)** in human VSMCs. The cells were pretreated with 10 μM CY-09 (an NLRP3 inhibitor) or Z-VAD-FMK (a pyroptosis inhibitor) for 2 h, then incubated with 200 μg/mL AGE for 48 h; **(D)** Immunofluorescence demonstrated that the pyroptosis inhibitor Z-VAD-FMK and the NLRP3 inhibitor CY-09 could reverse the effect of AGE on the phenotypic transformation of VSMCs. The cells were pretreated with 10 μM CY-09 (NLRP3 inhibitor) or Z-VAD-FMK (pyroptosis inhibitor) for 2 h, then incubated with 200 μg/mL AGE for 48 h; **(E)** RT-qPCR detected the expression of NLRP3 in human VSMCs. The cells were pretreated with 10 μM FPS-ZM1 (a RAGE inhibitor) or CCG-1423 (a RhoA inhibitor) or Y-27632 (a ROCK inhibitor) for 2 h, then incubated with 200 μg/mL AGE for 48 h. **(F)** A Transwell migration experiment demonstrated that pretreatment with the pyroptosis inhibitor Z-VAD-FMK, the NLRP3 inhibitor CY-09, the RAGE inhibitor FPS-ZM1, the RhoA inhibitor CCG-1423, and the ROCK inhibitor Y27632 could reverse the pro-migration effect of 200 μg/mL AGE on VSMCs. The cells were pretreated with 10 μM CY-09 (NLRP3 inhibitor), Z-VAD-FMK (pyroptosis inhibitor), FPS-ZM1 (RAGE inhibitor), CCG-1423 (RhoA inhibitor), or Y-27632 (ROCK inhibitor) for 2 h, then incubated with 200 μg/mL AGE for 48 h. Data are presented as the mean ± SD from three independent experiments and were analyzed with a one-way ANOVA followed by a Dunnett’s multiple comparisons test. Asterisks indicate significant differences (*P < 0.05, **P < 0.01, ***P < 0.001, ****P < 0.0001) between the treatment group and the control group. Pound signs indicate a significant difference (#P < 0.05, ##P < 0.01, ###P < 0.001) between the treatment group and the AGE group.

**FIGURE 3:**
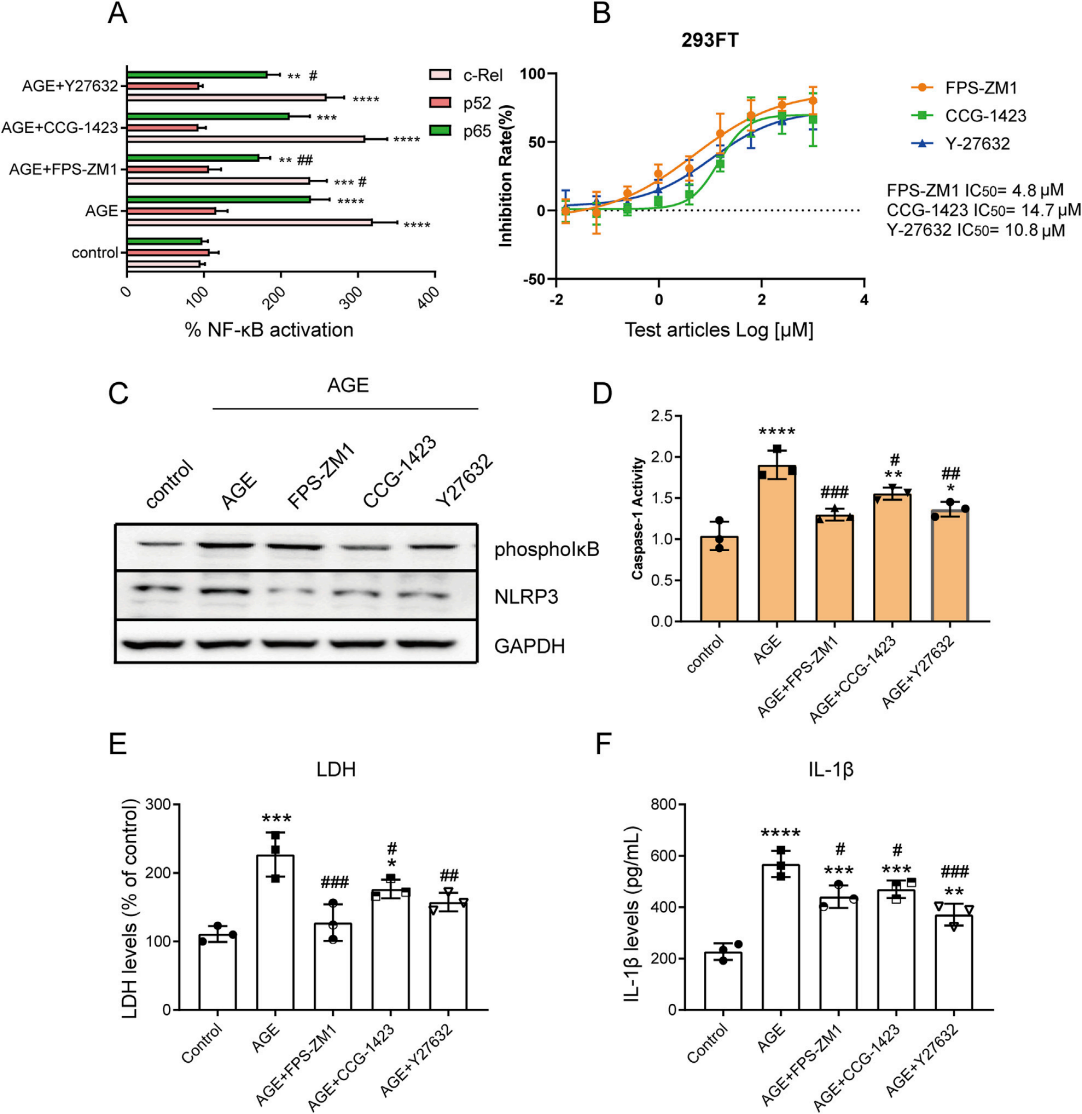
AGEs Activate the NF-κB Pathway and NLRP3 Inflammasome via the RAGE/RhoA/ROCK Pathway. **(A)** The TransAM NF-κB Transcription Factor Assay detected the effect of the RAGE/RhoA/ROCK inhibitor on the nuclear translocation of c-Rel, p52, and p65; **(B)** The NF-κB dual luciferase reporter gene system assessed the impact of RAGE/RhoA/ROCK inhibitors on AGE-induced NF-κB signaling; **(C)** Western blot analysis showed that AGEs regulate the expression of NLRP3 via the RAGE/RhoA/ROCK/NF-κB signaling pathway; **(D)** The effects of the inhibitors on caspase-1 activation were assessed using a caspase-1 assay kit; **(E)** An LDH release assay showed that AGE promoted VSMC cell pyroptosis, but it could be reversed by RAGE, RhoA, and ROCK inhibitors; **(F)** An ELISA assay demonstrated that AGE promoted the release of the inflammatory factor IL-1β through RAGE/RhoA/ROCK. Data are presented as the mean ± SD from three independent experiments and were analyzed with a one-way ANOVA followed by a Dunnett’s multiple comparisons test. Asterisks indicate significant differences (*P < 0.05, **P < 0.01, ***P < 0.001, ****P < 0.0001) between the treatment group and the control group. Pound signs indicate a significant difference (#P < 0.05, ##P < 0.01, ###P < 0.001) between the treatment group and the AGE group.

RT-qPCR and Western blot results demonstrated that AGEs enhance the expression of NLRP3 (Figures 2A, 3C). Furthermore, RT-qPCR, Transwell migration assays, and immunofluorescence analyses indicated that pre-incubation with the pyroptosis inhibitor Z-VAD-FMK and the NLRP3 inhibitor CY-09 could reverse the AGE-induced phenotypic transformation of VSMCs (from contractile to a migratory phenotype). Specifically, this treatment increased the expression of the contraction markers α-SMA and MYH11 while reducing the expression of the migration marker MMP-2 (Figures 2B–D). To exclude the possibility of drug-induced toxicity affecting cell behavior, cell growth was monitored under a microscope 48 h after treatment with AGEs, revealing no inhibition of cell growth or induction of apoptosis due to AGEs or inhibitor treatments (data not shown).

### AGE-induced transition of VSMCs to a migratory phenotype via the RAGE/RhoA/ROCK/NLRP3 pathway

RT-qPCR and Western blot analyses revealed that AGEs regulate NLRP3 expression through the RAGE/RhoA/ROCK pathway (Figures 2E, 3C). Additionally, Transwell migration assay results demonstrated that pre-incubation with the RAGE inhibitor FPS-ZM1, the RhoA inhibitor CCG-1423, and the ROCK inhibitor Y27632 could reverse the pro-migration effect induced by 200 μg/mL AGEs on VSMCs (Figure 2F).

### AGEs Activate the NF-κB Pathway and NLRP3 Inflammasome via the RAGE/RhoA/ROCK Pathway

TransAM NF-κB Transcription Factor Assay results demonstrated that AGEs activate the NF-κB signaling pathway via the RAGE/RhoA/ROCK pathway. Specifically, inhibition of RAGE/RhoA/ROCK suppressed the nuclear translocation of c-Rel and p65 without affecting p52 (Figure 3A). We then applied the NF-κB dual luciferase reporter gene system to assess the impact of RAGE/RhoA/ROCK inhibitors on AGE-induced NF-κB signaling. After 2 h of inhibitor treatment, 100 µg/mL AGEs were added, and the cells were incubated for an additional 48 h. The RAGE/RhoA/ROCK inhibitors significantly inhibited the NF-κB pathway, with IC_50_ values of 4.8, 14.7, and 10.8 µM, respectively (Figure 3B). A characteristic feature of inflammasome priming signals involves the activation of the NF-κB pathway to upregulate NLRP3. Western blot analysis revealed that AGEs regulate the expression of phospho-IκB and NLRP3 through the RAGE/RhoA/ROCK signaling pathway (Figure 3C). Activation of the NLRP3 inflammasome leads to Caspase-1 cleavage, triggering Caspase-1-dependent inflammatory cell death known as pyroptosis. Next, we investigated whether inhibitors of RAGE, RhoA, and ROCK can suppress the activation of caspase-1 triggered by AGEs. The cells were pretreated with the inhibitors for 2 h and then treated with AGEs for an additional 48 h. The effects of the inhibitors on caspase-1 activation were assessed using a caspase-1 assay kit. We found that the enhanced caspase-1 activity was significantly suppressed by inhibitors of RAGE, RhoA, and ROCK (Figure 3D). Levels of LDH release were used to assess pyroptosis induced by NLRP3 inflammasome activation, showing that AGEs promote VSMC cell pyroptosis, which can be reversed by inhibitors of RAGE, RhoA, and ROCK (Figure 3E). As caspase-1 activity is known to generate IL-1β, we also investigated the effect of inhibitors of RAGE, RhoA, and ROCK on IL-1β production. ELISA results indicated that AGEs enhance the release of the inflammatory factor IL-1β through RAGE/RhoA/ROCK signaling (Figure 3F).

### The AGE-RAGE signaling pathway is highly activated in αSMA-positive cells within abdominal aortic aneurysms featuring intraluminal thrombus (ILT)

**FIGURE 4:**
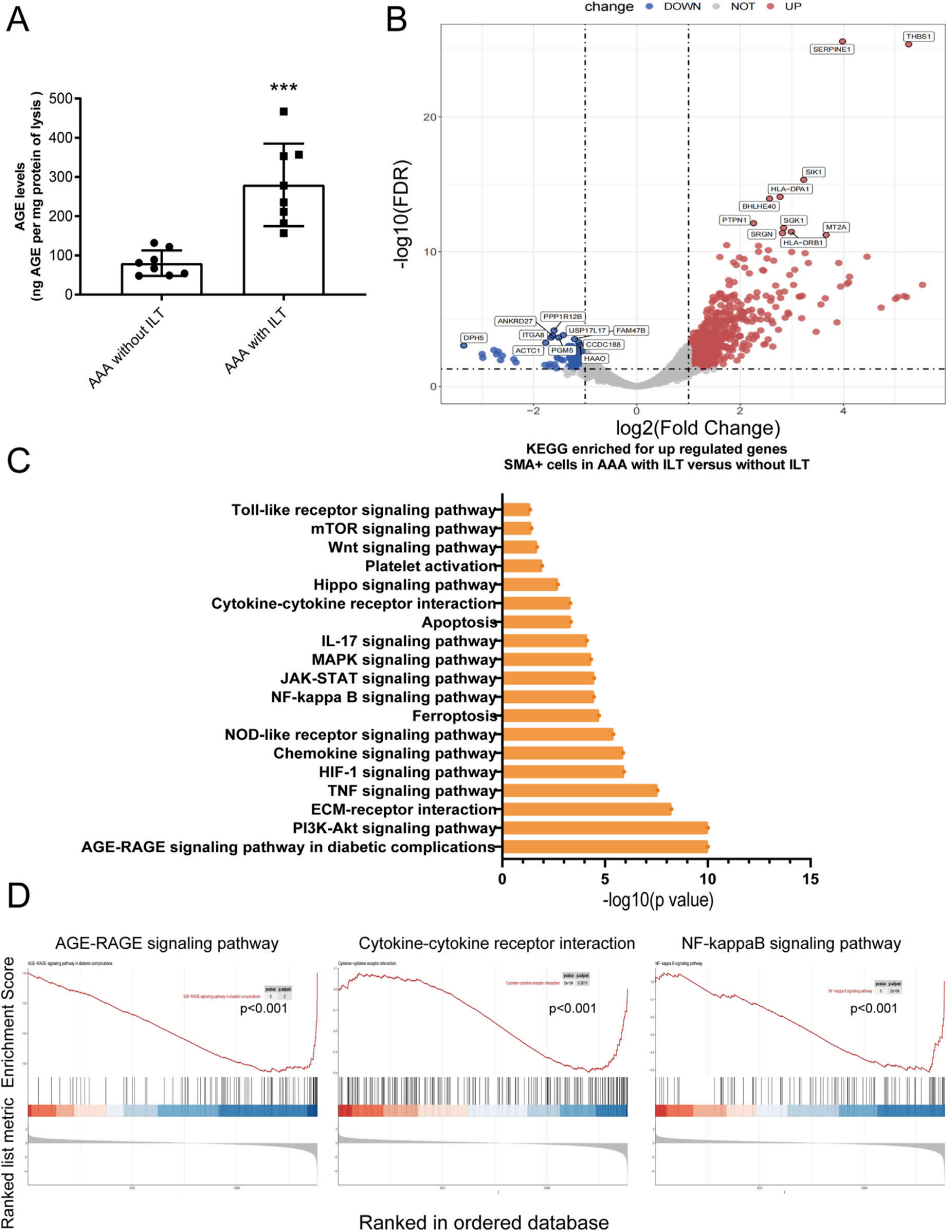
AGE-RAGE signaling pathway is highly activated in SMA-positive cells within abdominal aortic aneurysm featuring intraluminal thrombus. **(A)** An ELISA analysis detected an accumulation of AGEs in lysates of human aortic tissues. **(B–D)** Digital Spatial Profiling revealed strong enrichment of the AGE-RAGE signaling pathway in SMA-positive cells from AAAs with ILT compared to those without ILT. **(B)** The Volcano map of the gene expression profile; **(C,D)** the KEGG analysis of DEGs.

**FIGURE 5:**
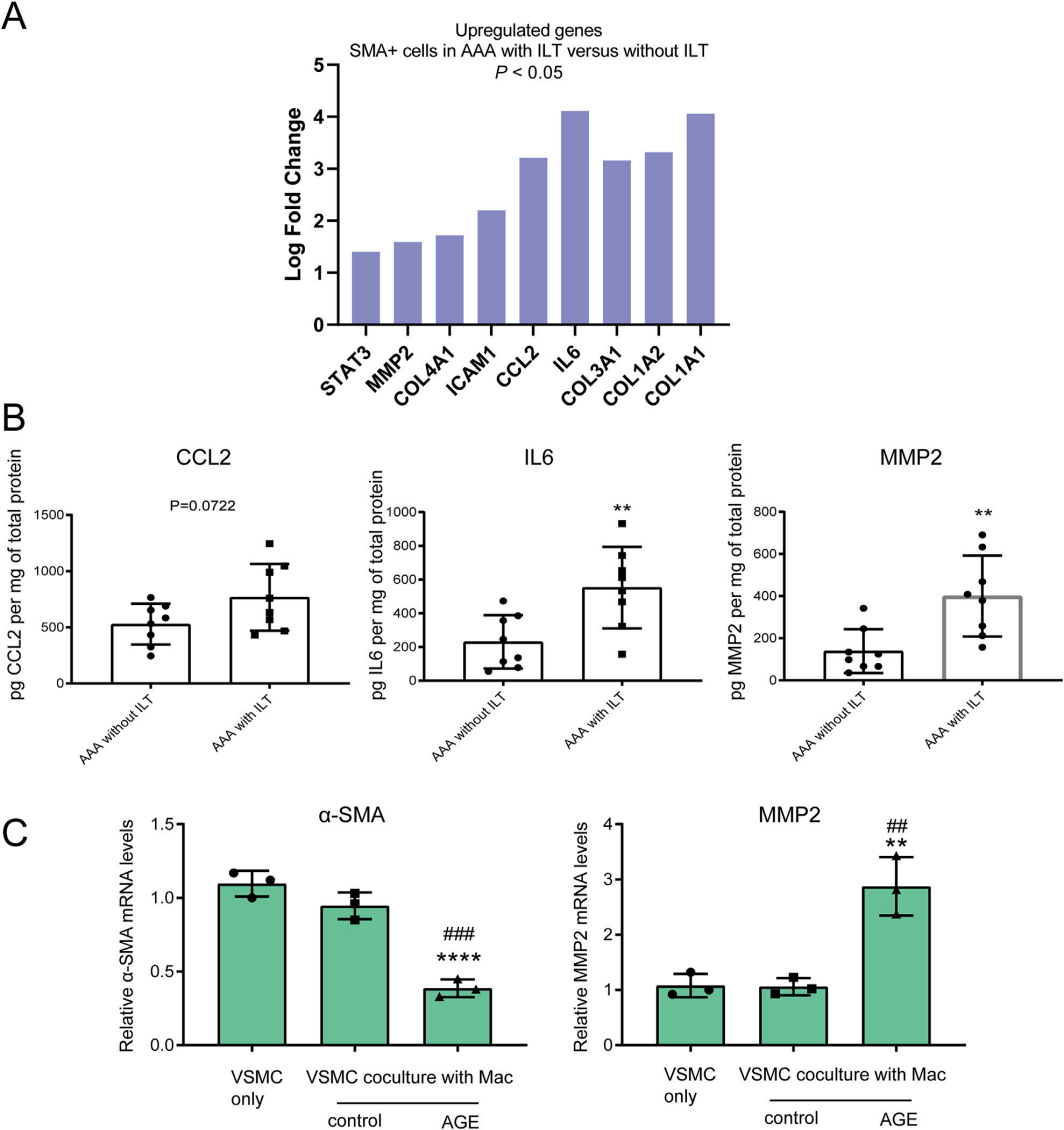
Inflammatory and migration phenotypes are featured in an abdominal aortic aneurysm with an intraluminal thrombus. **(A)** Using an adjusted p-value <0.05 and a |Log2Fold Change| > 1 as the criteria, we identified significantly differentially expressed mRNA, including elevated levels of critical genes involved in the AGE-RAGE signaling pathway; **(B)** ELISA assays confirmed increased expression of CCL-2, IL-6, and MMP-2 in aortas covered with ILT; **(C)** RT-qPCR detected the expression of the contraction marker α-SMA and the migration marker MMP-2 in human VSMCs after AGE treatment for 24 h in the co-culture system. Data are presented as the mean ± SD from three independent experiments and were analyzed with a one-way ANOVA followed by a Dunnett’s multiple comparisons test. ∼ versus VSMC only. Asterisks indicate a significant difference (**P < 0.01, ****P < 0.0001); ∼ versus VSMC co-culture with macrophages. Pound signs indicate a significant difference (##P < 0.01, ###P < 0.001).

Next, we investigated whether AGE expression differs between AAAs with ILT and AAAs without ILT. ELISA assays revealed a significant increase in AGE accumulation in human AAA tissues featuring ILT (Figure 4A). Using Digital Spatial Profiling, we found that several signaling pathways are enriched in SMA-positive cells (VSMCs) within AAAs with ILT compared to those without ILT (Figures 4B–D), including the AGE-RAGE signaling pathway, Cytokine-cytokine receptor interaction, and NF-kappaB signaling pathway, among others. We employed an adjusted p-value <0.05 and a |Log2Fold Change| > 1 as criteria to identify differentially expressed mRNAs. Among these, critical genes involved in the AGE-RAGE signaling pathway such as COL1A1, IL6, CCL2, MMP-2, and STAT3, among others, exhibited notably higher expression in SMA-positive cells of AAAs with ILT, showing statistical significance (Figure 5A; Supplementary Table S1). Additionally, ELISA confirmed elevated levels of IL-6 (inflammatory factor) and MMP2 (migration phenotype marker) in aortas covered with ILT (Figure 5B). Macrophage-like VSMCs are named for their similar surface markers and functions to macrophages. These cells contribute to chronic inflammation and may participate in the destruction of the aortic wall. Consistent with our expectations, the proportion of CD68+SMA+ double-positive cells (macrophage-like VSMCs) was significantly increased in AAAs with thrombus (data not shown). In AAAs with thrombus, we compared the molecular phenotypes of the CD68+SMA+ and CD68-SMA+ cell subpopulations. Notably, MMP-9 (logFC = 1.7) and IL-1β (logFC = 2.3) were significantly upregulated in AAAs with thrombus (data not shown). Further functional annotation through Gene Ontology (GO) and KEGG pathway enrichment analysis (using both ORA and GSEA methods) revealed significant enrichment in pathways related to inflammatory response regulation, cytokine-cytokine receptor interactions, NF-κB signaling, the AGE-RAGE signaling pathway, etc. (data not shown). *In vitro*, we co-cultured human macrophages (THP-1) and VSMCs. The macrophages were cultured in the lower chamber of a Transwell, while the VSMCs were cultured in the upper chamber. The results indicated that AGEs promoted the transformation of VSMCs into a migratory phenotype in the co-culture system, characterized by a decrease in α-SMA expression and an increase in MMP2 expression (Figure 5C).

### AGEs inhibit LPS-stimulated IL-6 and IL-1β secretion in human macrophage THP-1 cells

**FIGURE 6:**
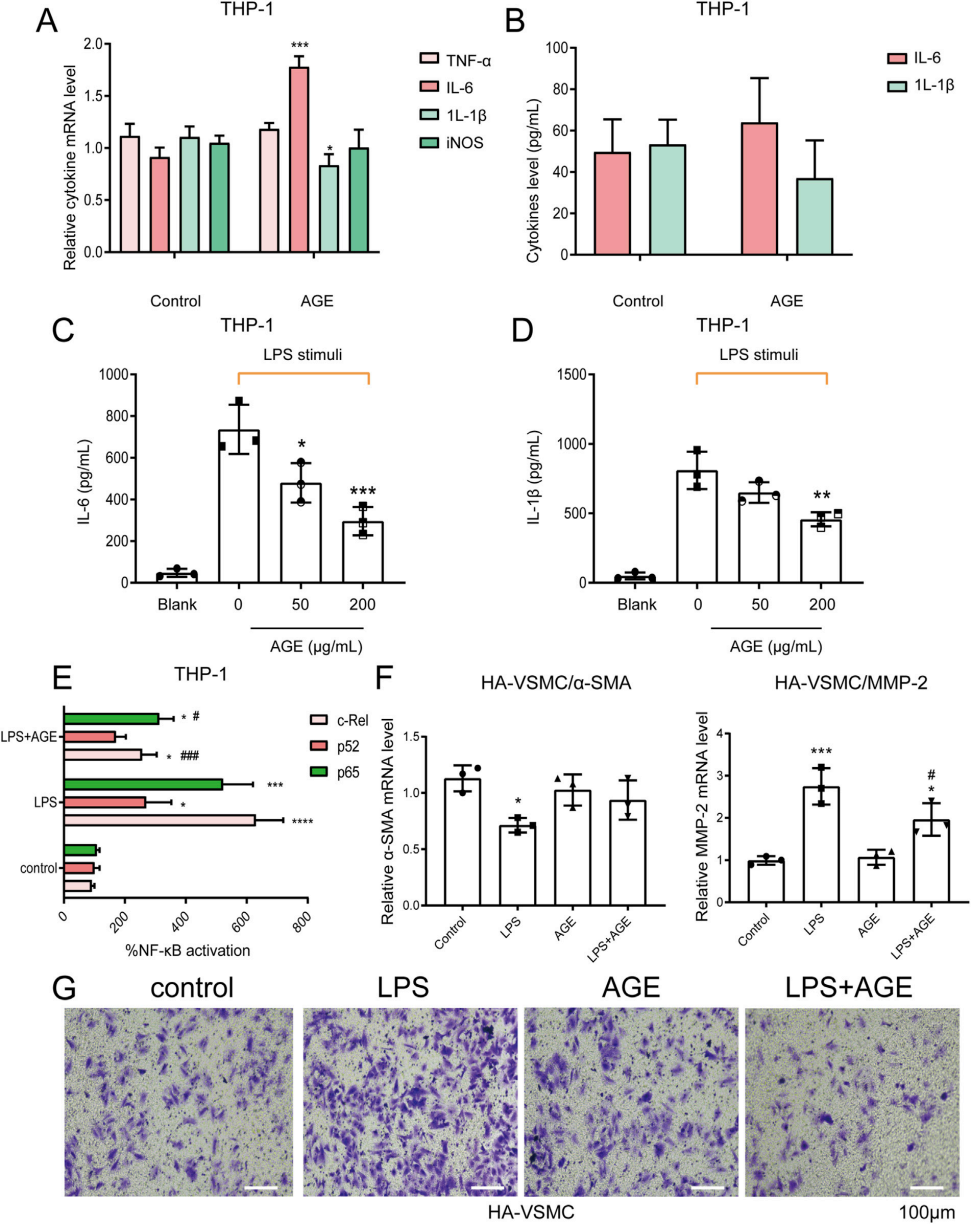
AGE Pretreatment Reverses the Effect of LPS-Stimulated Macrophages on VSMC Phenotypic Transformation. **(A,B)** qPCR **(A)** and ELISA **(B)** assays showed that in human macrophages THP-1, 200 μg/mL AGEs had little effect on the expression and secretion of cytokines TNF-α, IL-1β, and iNOS, and had a weak promoting effect on IL-6; **(C,D)** The ELISA assay demonstrated that pretreatment with AGE could inhibit the secretion of IL-6 and IL-1β induced by 1 μg/mL LPS stimulation; **(E)** The TransAM NF-κB Transcription Factor Assay showed that in human macrophage THP-1 cells, pretreatment with AGE could inhibit the NF-κB signaling pathway activated by LPS stimulation; **(F,G)** Human macrophage THP-1 cells were cultured in the lower layer. The cells were first pretreated with 200 μg/mL AGE for 18 h, and then stimulated with 1 μg/mL LPS for 4 h. After replacing the fresh medium, VSMCs were cultured in the upper layer of macrophages for 24 h before collection. RT-qPCR **(F)** detected the expression of the contraction marker α-SMA and the migration marker MMP-2 in human VSMCs; Transwell migration experiment **(G)** detected the migration of human VSMCs. Data are presented as the mean ± SD from three independent experiments and were analyzed with a one-way ANOVA followed by a Dunnett’s multiple comparisons test. Asterisks indicate significant differences (*P < 0.05, **P < 0.01, ***P < 0.001) between the treatment group and the control group. Pound signs indicate a significant difference (#P < 0.05) between the treatment group and the LPS group.

Vascular inflammation plays a crucial role in the pathogenesis of vascular diseases. Inflammatory mediators originating from inflammatory cells (such as macrophages) within vascular lesions promote the development of stenotic lesions through the proliferation and migration of vascular smooth muscle cells [22, 23]. Macrophages exhibit both pathogenic and protective functions in the pathophysiology of abdominal aortic aneurysms by participating in inflammation. Results from RT-qPCR and ELISA demonstrated that in human macrophage THP-1 cells, treatment with 200 μg/mL AGEs had minimal effect on the expression and secretion of the cytokines TNF-α, IL-1β, and iNOS, with a modest stimulatory effect observed on IL-6 (Figures 6A,B), which is the opposite of what has been implicated in VSMCs. Moreover, unexpectedly, ELISA results indicated that pre-incubation with AGEs could attenuate the secretion of IL-6 and IL-1β induced *by LPS stimulation* (Figures 6C,D). Experimental findings from TransAM NF-κB Transcription Factor Assays showed that pre-incubation with AGEs inhibited the NF-κB signaling pathway activated by LPS in THP-1 cells (Figure 6E).

### AGE pretreatment reverses the effect of LPS-stimulated macrophages on VSMC phenotypic transformation

THP-1 cells were cultured in the lower layer of a co-culture system. Initially, the cells were pretreated with 200 μg/mL AGEs for 18 h, followed by stimulation with 1 μg/mL LPS for an additional 4 h. After replacing the medium, vascular smooth muscle cells were cultured in the upper layer of the co-culture system. RT-qPCR results revealed that AGE pretreatment reversed the inhibitory effect of LPS-stimulated macrophages on the contraction phenotype of VSMCs. Specifically, the expression of the contraction marker protein α-SMA increased, while the expression of the migration marker protein MMP2 decreased (Figure 6F). Furthermore, Transwell migration assay results demonstrated that AGE pretreatment attenuated the promotional effect of LPS-stimulated macrophages on VSMC migration (Figure 6G). In summary, AGEs exert markedly different effects on macrophages compared to VSMCs, which highlights the cell-type-specific immunomodulatory effects of macrophages in AAAs. This refers to the two opposing or complementary roles that macrophages may play in different environments or contexts. For example, they can either promote or inhibit inflammation and immune responses.

## Discussion

Vascular smooth muscle cells (VSMCs) are one of the most important components of the human aortic wall, and their normal structure and function are essential to the intact biomechanical properties of the aortic wall [24]. Many damaging factors, such as hemodynamic changes and the inflammatory response, can stimulate VSMCs to undergo phenotypic transformation [25, 26]. VSMCs play a pivotal role in the pathogenesis of vascular diseases due to their ability to transition from a contractile to a synthetic phenotype. This phenotypic switch enables VSMCs to proliferate, migrate, and produce pro-inflammatory cytokines, contributing significantly to vascular disease processes [27].

Zhang et al. [28] demonstrated significantly higher levels of advanced glycation end products (AGEs) in the aortas of abdominal aortic aneurysm (AAA) patients compared to healthy subjects using immunohistochemistry and ELISA. They also found elevated expression levels of the receptor for AGEs (RAGE) in human and mouse AAA tissues compared to normal aortic tissues. Prasad et al. showed a positive correlation between serum AGE levels and IL-1β, IL-6, and MMP-2 levels in patients with thoracic aortic aneurysms (TAA). Additionally, AGE-RAGE stress in TAA patients was positively correlated with MMP-2 levels [29]. Our study revealed a significant accumulation of AGEs in human AAA specimens, especially those covered with intraluminal thrombus (ILT). To avoid the influence of antiplatelet therapy on AGE levels, we analyzed clinical sample data that did not include antiplatelet therapy, and our conclusions remained consistent. Furthermore, the AGE-RAGE signaling pathway was highly enriched in smooth muscle actin (SMA)-positive cells (VSMCs) in AAAs with ILT compared to AAAs without ILT. Before this study, the molecular mechanisms by which the AGE-RAGE axis promotes inflammatory responses and migratory phenotypes in AAA, particularly in AAA with ILT, were not well understood.

The Rho family of small GTPase proteins, particularly RhoA, plays a critical role in regulating various cellular processes. RhoA acts through its effector Rho kinase (ROCK) to modulate the organization of the actin cytoskeleton, thereby influencing cellular functions such as contraction, motility, proliferation, and apoptosis. Zhao et al. [30] showed that high glucose stimulation increases the permeability of human umbilical vein endothelial cells and upregulates myosin light chain (MLC) phosphorylation. Rao et al. [31] reported that the RhoA/ROCK pathway is crucial for regulating cell migration and that ROCK inhibitors suppress the release of adhesion molecules such as intercellular cell adhesion molecule-1 (ICAM-1) and monocyte chemoattractant protein-1 (MCP-1). Based on these findings, we hypothesized that the RhoA/ROCK pathway might serve as a key downstream signaling pathway for AGEs-RAGE in regulating inflammatory responses and phenotypic transformation. Our study demonstrated that pretreatment with the RAGE inhibitor FPS-ZM1, the RhoA inhibitor CCG-1423, and the ROCK inhibitor Y27632 could reverse the pro-migration effect induced by AGEs on human VSMCs.

The interaction between advanced glycation end products and their receptor RAGE leads to the generation of reactive oxygen species (ROS) by activating nicotinamide adenine dinucleotide phosphate (NADPH) oxidase [32], which in turn activates the transcription factor NF-κB [33], thereby inducing an inflammatory response [34]. Canonical activation of the NLRP3 inflammasome involves an initial signal regulated by NF-κB to transcribe NLRP3 and pro-IL-1β, followed by a second signal to assemble the NLRP3 inflammasome. This assembly facilitates the cleavage of caspase-1 and the subsequent maturation and secretion of IL-1β [35]. Song et al. demonstrated that the RAGE/NF-κB pathway can activate the NLRP3 inflammasome, leading to the maturation of IL-1β in nucleus pulposus cells [36]. Our research has demonstrated that AGEs regulate the expression of NLRP3 through the RAGE/RhoA/ROCK/NF-κB signaling pathway and pretreatment of VSMCs with the pyroptosis inhibitor Z-VAD-FMK and the NLRP3 inhibitor CY-09 for 24 h can reverse the effects of AGEs on the phenotypic transformation of VSMCs.

In addition to RAGE, AGEs bind to and signal through TLR4 [37], which acts as a receptor for multiple pathogen-associated molecular patterns (PAMPs), including lipopolysaccharides from Gram-negative bacteria (LPS), which can induce inflammatory effects [38]. AGEs interacting with RAGE or TLR4 can stimulate multiple transcription factors; among these, NF-κB is crucial for M1 polarization and mediates pro-inflammatory responses in macrophages and other cell types [39]. In our study using human macrophage THP-1 cells, we observed that a high concentration of AGEs had a minimal impact on the expression and secretion of the cytokines TNF-α, IL-1β, and iNOS, and had a modest promoting effect on IL-6. Interestingly, AGEs inhibited the secretion of IL-6 and IL-1β induced by LPS stimulation in THP-1 macrophages. Furthermore, a TransAM NF-κB Transcription Factor Assay demonstrated that AGEs inhibited the NF-κB signaling pathway activated by LPS in THP-1 macrophages.

Multiple studies have indicated the presence of macrophages in the walls of aneurysmal aortas, as observed in both human aortic sections and animal models of abdominal aortic aneurysms [40–42]. The accumulation of macrophages in the aneurysmal aortic wall plays a vital role in driving inflammation, extracellular matrix degradation, and tissue remodeling during the healing process. Numerous studies have provided *in vitro* evidence suggesting potential crosstalk between macrophages and smooth muscle cells via cytokine secretion, including PDGF, IL-6, TNF-α, and MCP-1 [43]. Human macrophages strongly induce VSMC apoptosis through direct cell-to-cell interactions mediated by Fas/Fas-L, which contributes to plaque rupture [44]. Additionally, another study demonstrated that macrophages promote VSMC apoptosis and influence VSMC phenotype switching through a circRNA-mediated mechanism [45]. In our previous study, we demonstrated that overexpression of hsa_circ_0087352 in macrophages induced smooth muscle cell apoptosis in a co-culture system, likely due to the release of pro-apoptotic cytokines such as IL-6, TNF-α, and IL-1β [19]. Initially, we speculated that macrophages pretreated with AGEs might promote the transition of VSMCs to a migratory phenotype. Notably, our data demonstrate that AGE pretreatment attenuates LPS-induced proinflammatory cytokine production and NF-κB activation in macrophages. This immunomodulatory effect aligns with the established mechanism reported by Son et al. [46], in which AGEs suppress NLRP3 inflammasome assembly and TLR signaling via non-RAGE pathways, specifically inhibiting M1 polarization. Then, in our experimental setup, human macrophage THP-1 cells were cultured in the lower chamber of a coculture system. We pretreated these cells with 200 μg/mL AGEs for 18 h, followed by stimulation with 1 μg/mL LPS for 4 h. After replacing the medium with fresh medium, VSMCs were cultured in the upper chamber above the macrophages. Unexpectedly, the results showed that pretreatment with AGEs reversed the pro-migration effect of LPS-stimulated macrophages on VSMCs. While some previous studies have reported that AGEs do not significantly promote cytokine production [46–48], other findings suggest that AGEs can indeed increase cytokine secretion [49–51]. Further detailed studies are needed to fully understand the complex role of AGEs in the macrophage-mediated inflammatory responses in abdominal aortic aneurysms.

In diabetic vascular disease—where AGE accumulation is pronounced—clinical trials of AGE-RAGE inhibitors (e.g., Alagebrium) have demonstrated reduced vascular stiffness and improved endothelial function [52, 53]. While these findings highlight the pathway’s biological relevance, they do not directly support the therapeutic efficacy in AAA. Mechanistically, inhibition of RAGE or downstream RhoA/ROCK signaling may modulate processes implicated in AAA pathogenesis, such as VSMC phenotypic transformation. However, the absence of *in vivo* AAA outcome data precludes conclusions about clinical benefits. Substantial challenges remain, including: a) the complexity of AGE formation and cross-tissue involvement, raising concerns about therapeutic specificity; b) the potential for off-target effects when inhibiting RAGE/RhoA/ROCK in non-vascular tissues; c) a lack of evidence regarding dosing, timing, or patient selection in AAA contexts—particularly in cases with comorbidities such as diabetes; and d) the critical need for long-term safety/efficacy studies in AAA-specific models before considering clinical translation.

### Limitations of this study

First, the current research primarily relies on *in vitro* models, which may not fully capture the complexity of AAAs in a living organism. Thus, future studies are critical for validating the therapeutic effects of AGE-RAGE signaling pathway inhibitors in animal models of AAAs, especially those with thrombus.

Second, our study used the HA-VSMC cell line, which was derived from an 11-month-old female donor, representing a significant biological mismatch with the predominant elderly male demographic in AAAs. While this model provides standardized insights into VSMC behavior, it fails to capture critical age- and sex-specific pathways that influence AAA pathogenesis, such as senescence-associated secretory phenotypes and androgen-mediated signaling. Future studies should prioritize primary VSMCs from age- and sex-matched AAA patients.

Third, our use of PMA-differentiated THP-1 macrophages represents a significant limitation in our ability to model human AAA pathophysiology. While this system revealed that AGE pretreatment attenuated LPS-induced IL-6/IL-1β release and reduced macrophage-driven VSMC migration in co-culture, PMA differentiation fails to replicate critical aspects of ILT-associated macrophage heterogeneity in AAAs. Consequently, our observed anti-inflammatory effect of AGEs on LPS-challenged macrophages may not translate to the complex AAA microenvironment, where thrombus hypoxia, erythrocyte lysis, and matrix remodeling create unique signaling milieus. Future studies must validate these interactions using the following: a) macrophages isolated from human AAAs; b) ILT-integrated AAA animal models with single-cell RNA sequencing; c) spatial multi-omics to resolve AGE-RAGE effects on *bona fide* disease-associated macrophage subsets.

Fourth, the small cohort size reduced statistical power for subgroup analyses and precluded the formal correlation of AGE levels with clinical severity markers (e.g., aneurysm size/growth rate). Additionally, while our aorto-iliac occlusive disease controls provide age- and comorbidity-matched comparisons for AAA patients, they do not represent true healthy aortic biology. As explicitly demonstrated in the landmark study by Zhang et al. [28] comparing AAA to autopsy-derived normal aortas, the use of truly healthy controls remains the gold standard for establishing disease-specific biomolecular changes. We emphasize the need for cautious interpretation until independent validation in larger cohorts is performed.

Fifth, while this study establishes cell-type-specific immunomodulatory effects of AGEs - activating RAGE-dependent pro-inflammatory pathways in VSMCs versus suppressing NLRP3 inflammasome activation in macrophages via non-RAGE mechanisms (Son et al. [46]) - key mechanistic aspects remain unresolved. Specifically, the identity of the alternative receptors mediating AGE immunosuppression in macrophages (e.g., scavenger receptors) remains undefined in our experimental system. Future investigations should: a) employ receptor-knockout macrophages to validate non-RAGE pathways; b) develop spatial-omics approaches to map AGE-receptor interactomes across vascular niches; and c) define temporal thresholds for the transition from AGE-mediated immunosuppression to inflammation.

**FIGURE 7:**
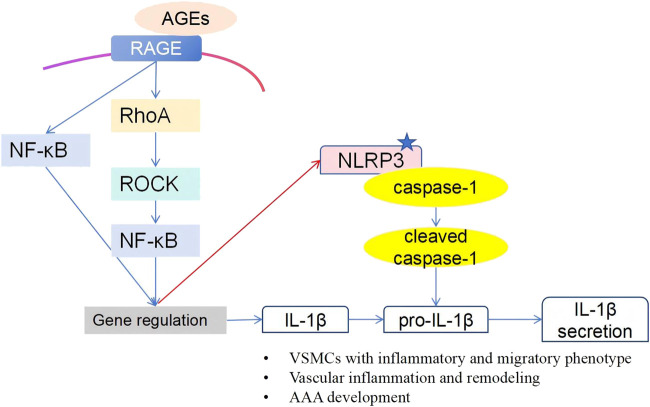
Schematic overview of the AGE-induced phenotypic switch in human VSMCs. Our research demonstrated that, in human VSMCs, AGEs activate the NLRP3 inflammasome through the RAGE/RhoA/ROCK/NF-κB signaling pathway, inducing an inflammatory and migratory phenotype.

Collectively, our data reveal cell-type-specific modulation by AGE-RAGE signaling: promoting inflammatory pathways in VSMCs (e.g., ROCK/NF-κB/NLRP3; Figure 7) while attenuating inflammatory responses in macrophages. This observed dichotomy advances our understanding of metabolic influences in AAAs, but it requires rigorous causal validation in disease-relevant models before considering clinical applications.

## Data Availability

The datasets presented in this article are not readily available because the authors are affiliated with Changhai Hospital, an institution designated with a national security classification level, and institutional policy strictly prohibits the public deposition of raw research data. Requests to access the datasets should be directed to the corresponding author, CS.
