# Supplementary material for: Mechanisms of AGE-induced VSMC phenotypic switching and macrophage modulation in human abdominal aortic aneurysms
Source: Exp Biol Med (Maywood). 2025 Aug 7;250:10527. doi: 10.3389/ebm.2025.10527 (PMC12367564; doi:10.3389/ebm.2025.10527)
Supplement: Supplementary file 2 [file DataSheet1.docx]

**Supplementary Figures**

Supplementary Figure 1


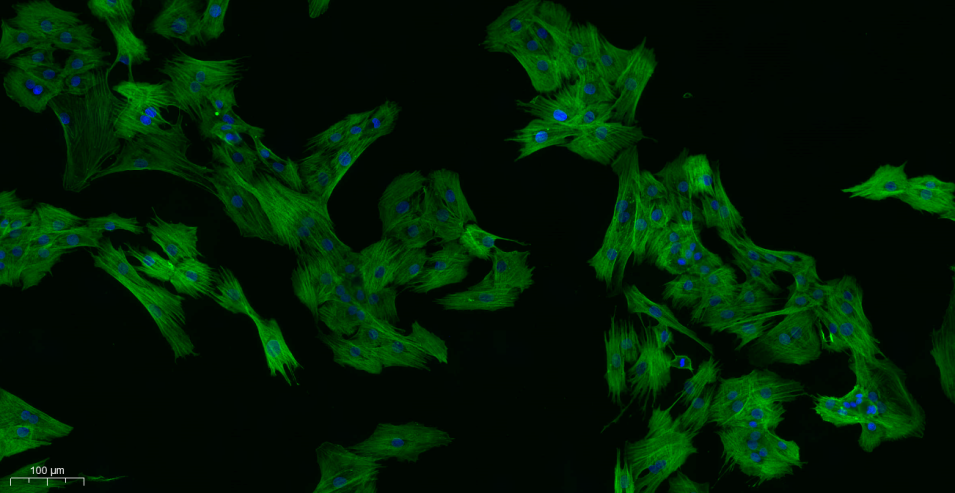


HA-VSMC cells were incubated with Anti-alpha smooth muscle Actin antibody (abcam, Cat. No. ab7817), with green fluorescence indicating α-SMA positivity.

Supplementary Figure 2

Effect of AGE on the activation of NF-κB signaling pathway in 293T Cells. The highest concentration of AGE was 10 mg/mL, with 4-fold serial dilutions, resulting in 9 concentration gradients. The cells were treated for 24 hours.
